# Qualitative Investigation into Pre- and Post-Natal Experience of Parents of Triplets

**DOI:** 10.1007/s10826-021-02200-1

**Published:** 2021-12-18

**Authors:** Yasuhiro Kotera, Greta Kaluzeviciute, Laura Bennett-Viliardos

**Affiliations:** 1 University of Nottingham, Jubilee Campus, Nottingham, NG7 2TU UK; 2 University of Derby, Kedleston Road, Derby, DE22 1GB UK

**Keywords:** Triplets, Parental wellbeing, Multiple birth, Qualitative research, Thematic analysis

## Abstract

Although parents of triplets experience substantial mental distress, research about this increasing population has primarily focused on physical health risks of triplets and mothers, failing to capture the subjective wellbeing of parents. Accordingly, this study aimed to understand first-hand experience of parents of triplets, using thematic analysis of semi-structured interviews participated by eight parents (four couples: Age M = 48.63, SD = 10.61 years). Six themes were identified: (1) Negative and (2) Positive experiences of raising triplets prenatally and postnatally, (3) Social, psychological, and material support, (4) Experiences and challenges specific to mothers and (5) fathers, and (6) Advice for future parents. These themes suggest that being reassured and accepting support from others are particularly essential in reducing stress and anxiety. Self-compassion interventions were recommended to support the wellbeing of parents of triplets. Our findings will help parents of triplets, their social circles, and healthcare workers to develop effective approaches to reduce the mental health difficulties that this under-researched population experiences.

## Highlights

- Parents of triplets experience increased anxiety and stress related to triplets’ and mothers’ health and financial concerns.
- Parents’ positive experiences included a sense of having a big family and of feeling magical to encounter the unusual phenomena.
- Mothers’ challenges were internal (health-related), while fathers’ were external (stability of job and finances).
- Being reassured and accepting support from others are particularly essential for reducing stress and anxiety in this population.

The occurrence of higher order gestations or multiple births, consisting of three or more foetuses, has been rapidly increasing over the past three decades in developed countries (Ferraretti et al., 2012; Habbema et al., 2009; Pison and D’Addato, 2006). This increase is correlated with medical advancements such as in-vitro fertilisation (IVF) and assisted reproductive techniques (ART) (Ferraretti et al., 2012; Habbema et al., 2009). The growing tendency towards delaying childbearing (Martin et al., 2018; Pison and D’Addato, 2006) or difficulties in conceiving have led to an increased use of fertility treatments and partially explains the trend of triplet conceptions (Jenkins & Coker, 2010). Even without these interventions, delayed childbearing alone was associated with an increased likelihood of multiple births (Martin et al., 2012). Historically, the rate of spontaneous triplet gestations was around 1 in 8000 pregnancies (Benirschke & Kim, 1973). With the advanced use of multiple embryo transfers during ART, in order to improve the chance of a liveborn pregnancy, the rate of triplet pregnancies raised exponentially by over 400% in the United States of America (Martin et al., 2018). The rates peaked around 1998 when around 1 in 515 births were triplet and higher order pregnancies (Martin et al., 2018). Following the introduction of national guidelines reducing multiple embryo transfers during ART, these figures began to decline (Martin et al., 2016). In the United Kingdom, the most recent multiple birth statistics available reported that 0.2 out of 1000 births were triplets (Office for National Statistics, 2018). Although the studies have noted IVF and ART as the probable cause for the rates of higher order gestation, there is a lack of research available on spontaneous triplet pregnancies.

It is well established that triplet pregnancies are high risk, with an increased risk of maternal and infant morbidity and mortality (Bricker, 2014). An increased number of foetuses increases the risk of adverse neonatal outcomes, primarily due to preterm deliveries (before 32 weeks from gestation), which occurs in approximately 90% of triplet deliveries (Blumenfeld et al., 2008; Tucker & McGuire, 2014). Premature labour in triplet pregnancies is around three times higher than twins, and 24 times higher than singletons (Martin et al., 2012). Other factors related to adverse outcomes for triplet infants are associated with foetal growth restriction, foetal abnormality and complications due to shared placentation (Pharoah & Cooke, 1996). It has also been found that the risk of cerebral palsy is 24 times higher among triplets, compared to six times higher in twins (Pharoah & Cooke, 1996). In multiple pregnancies, mothers are at a greater risk of experiencing a miscarriage, hypertensive disorders, anaemia, haemorrhage, delivery by caesarean section, and post-natal depression (National Collaborating Centre for Women’s and Children’s Health, 2011). The rate of maternal mortality in multiple gestations is twice that of singleton pregnancies (Lewis, 2004) and can often be overshadowed by concerns for the foetuses (Bricker, 2014; Senat et al., 1998). Despite the risks associated with higher order pregnancies, treatment for premature babies with respiratory issues has advanced considerably (Weismann et al., 2013). Further, prenatal treatment has also improved significantly, and most women will have access to antenatal care in specialised high risk pregnancy facilities with regular follow up support (Weismann et al., 2013). However, the improvements in healthcare and the subsequent decline in mortality risk generates an increasing need for medical and social services for families and infants of multiple births (Jenkins & Coker, 2010; Kiely Kleinman & Kiely, 1992). Although there have been significant medical developments in reducing mortality and morbidity for premature infants, the clinical outcomes of triplets have not seen much improvement in the last three decades (Weissman et al., 2013).

The obstetric and gynaecological risks of multiple pregnancies, alongside the neonatal outcomes, have been well documented in the literature (Bricker, 2014; Roca-de Bes et al., 2011; Martin et al., 2012); however, there is also a psychosocial impact to consider (Weismann et al., 2013). For those who used ART and IVF, it is likely that families with triplet pregnancies experienced difficulties with infertility, which is often associated with anxiety and emotional distress (Baor & Blickstein, 2005). As there are significant risks involved in a triplet pregnancy, the decision to continue a pregnancy can result in short-term and long-term stress and anxiety (e.g., decision to decline doctor’s suggestions for selective reduction; Mhatre & Craigo, 2020). Subsequently, this heightened anxiety highlights a need for clinicians to provide specific mental health support and information during pregnancy (Bricker, 2014). Roca-de Bes et al., (2011) emphasise the need for psychological therapy during higher order pregnancies and argue that counselling should also be provided for the parents.

As well as the psychological impact of triplet births, there are a number of other challenges associated with raising triplets including economic stressors, quality of life, marital adjustment, and psychological disorders (Roca-de-Bes et al., 2011). A common cause of marital problems is related to the financial pressures involved in raising triplets (Jena et al., 2011). Evidence suggests that families raising triplets report that they have needed additional help and financial support for childcare, household costs and medical expenses (Ellison et al., 2005; Strauss et al., 2008). Compared to parents of singleton births, families of triplets are three times more likely to have encountered challenges in meeting their basic needs (Ellison et al. 2005; Strauss et al. 2008). Furthermore, in order to reduce childcare costs, it is common for one parent to not return to the workforce after birth (Strauss et al., 2008). Although this decreases childcare costs, the reduced income can significantly contribute to the household financial strain (Glazebrook et al., 2004; Strauss et al., 2008). In addition to the economic burden of caring for triplets, parents often experience considerable physical and emotional exhaustion (Mhatre & Craigo, 2020). It has been suggested that mothers of triplets are more likely, than mothers of singletons, to experience feelings of tiredness, stress and depression (Ellison et al., 2005). Moreover, it has been reported these symptoms can often persist long after the neonatal period into childhood (Garel et al., 2008). Furthermore, these mental health difficulties have been exacerbated by the COVID-19 pandemic. Though not triplet-specific, 79% of parents of multiple births reported an increase in stress and anxiety during the pandemic (Twins Trust 2020). In previous research exploring how parents cope with triplets, parents described feeling *‘out of control of the care of the babies’* due to the fact that the pregnancy was highly monitored by medical professionals (Jenkins & Coker, 2010 p.172). Parents also stressed that they felt *‘overwhelmed and frightened’* by having to learn to manage with three babies (Jenkins & Coker, 2010 p.173). Parents highlighted a need to hear the stories of other triplet parents to share experiences and draw on their wisdom (Jenkins & Coker, 2010). These findings suggest a need to understand the experience of parents of triplets.

The high instances of stress, exhaustion, and marital difficulties reported by mothers of triplets can exacerbate difficulties in forming a bond with each child (Akerman, 1999; Albrecht & Tomich, 1996; Leonard, 1998). As triplet families can be viewed as rare or unusual, parents have reported feeling restricted when it comes to seeking help from family or community members (Beit-Hallahmi & Paluszny, 1974; Scheinfeld, 1967). Further, mothers raising multiples have reported that their experiences of stigma and stress were concerning issues (Ellison & Hall, 2003). As it is noted that triplet births are considerably more stressful than that of twins or singletons, Feldman and Eidelman (2004 p.1775) argue that a triplet birth can compromise the mother’s ability to provide *‘sensitive mothering’* to each child and mothers have reported having limited energy to form an emotional bond with each infant (Bryan, 1992). From an attachment perspective, reduced maternal sensitivity could have an impact on a child’s development (Ainsworth et al., 1978; Bowlby, 1969). There are limited studies available on the cognitive development of triplets (Feldman et al., 2004). However, a study by Feldman and Eidelman (2009) found that, compared with singletons and twins, triplets displayed lower cognitive performance at 6, 12 and 24 months. Despite this, by five years of age, these differences decreased. In addition, lower social interactions observed in triplets at two years, were no longer present in triplets at five years of age. It was also noted that difficulties in maternal adjustment, experienced by mothers of triplets, began to decrease as the children reached five years of age. Research on the development of triplets, during adolescence, found that parents reported significantly less behavioural problems in triplets compared with the families of singletons (Natalucci et al., 2012). Furthermore, a triplet child will typically spend as much time with their siblings as they spend with the main caregiver (Feldman & Eidelman, 2004) and triplets often develop a strong lifelong bond (Twins Trust, 2021).

## Study Aims

Although there are many studies assessing the external factors such as risks, outcomes and management of triplet pregnancies (Mhatre & Craigo, 2020), there is a paucity of research available on the internal experience of parents of triplets, following from pre- to post-natal phases. Accordingly, this study aims to understand the first-hand experience of parents of triplets through qualitative investigation. Our analysis considers challenges, resources, positive impacts, and advice for birth and child-raising of triplets.

## Methods

### Research Design

Thematic analysis of in-depth semi-structured interviews with eight parents of triplets who had given birth at least one year ago (four male-female couples; Age M = 48.63, SD = 10.61, Range 32–60 years old; Age of triplets at the time of the study = 19, 3, 27 and 25 years old; 3 couples lived in the UK and 1 in Japan; see/Table 1). Two triplet families also have an older sibling (a sister), while the two remaining triplet families have no other siblings. All of them had natural birth. Consolidated criteria for reporting qualitative research were followed in this study (Tong et al., 2007).

**Table 1:** List of participants

Couple	Participant #	Gender	Age (Years)	Residence	Age of Triplets (Years at the time of the study)	Other Sibling(s)
Couple A	1	Female	51	UK	19	1 older sister by 4 years
	2	Male	55	UK		
Couple B	3	Female	32	Japan	3	1 older sister by 3 years
	4	Male	33	Japan		
Couple C	5	Female	58	UK	27	None
	6	Male	60	UK		
Couple D	7	Female	50	UK	25	None
	8	Male	50	UK		

### Procedure and Analysis

Ethical approval of this study was granted by the university’s research ethics committee (No. 260219YK). Purposive and snowball sampling techniques were used to recruit the participants: an initial announcement about the study was shared through the lead author’s social media accounts. The parents who responded to the announcement received an invitation to the study and a consent form, which explains the rationale and details of the study. Participants who agreed to take part in the study completed the informed consent form electronically, which was sent back to the researchers.

In order to gather detailed information and allow participants to convey their experiences of parenthood, we employed the semi-structured interview method (Woods, 2011). Semi-structured interviews fall between standardised, close-ended surveys and free form, open–ended sessions with groups (Adams, 2015). They are particularly suitable for research studies that tackle complex topics and phenomena, which means that researchers may wish to pose follow-up queries, while participants may want to provide additional information that is relevant for the study but does not necessarily respond to template questions. Since experiential accounts on parents undergoing multiple births have not been qualitatively examined to date (Bricker, 2014), we thought it was important to allow space for probing, open-ended questions that would in turn yield richer data material.

Pre-designed interview questions were formed to provide some guidance and sent to all participants in advance of the interview. Our questions focused on parents’ pre- and post-natal experiences of having triplets, the challenges and positive impacts of parenting, advice for future parents of triplets, other people’s reactions (Appendix 1). Although we interviewed parent couples, we conducted separate interviews with each father and mother to observe variations in idiosyncratic experiences specific to different parent roles. Since our participants were based at different countries, we conducted video interviews via Skype. The interviews were recorded (with the participants’ prior consent) and transcribed verbatim, which were later confirmed for accuracy by the participants.

We used the thematic analysis method to analyse the interview data. Thematic analysis is a method used for systematic identification and organisation of patterns of meaning (themes) across a dataset (Braun & Clarke, 2012). It is suitable for research studies that seek to make sense of shared meanings and identify unique and/or divergent idiosyncratic experiences. Crucially, thematic analysis does not merely focus on what is common; instead, themes are determined in terms of their importance in relation to the researched phenomenon. In order to determine the relevant themes in the interview data, we conducted thematic analysis in the following order: (i) Familiarisation, (ii) Generating initial codes, (iii) Searching for themes, (iv) Reviewing themes, and (v) Defining and naming themes (details on each process are available below) (Braun & Clarke, 2006).

Y.K., a mental health researcher and practitioner, interviewed all couples and transcribed the interview data. G.K., a qualitative mental health researcher analysed the transcribed data using thematic analysis. Y.K. and L.B.V., a mental health researcher and practitioner, reviewed the analysis. Although we acknowledge, with Braun and Clarke (2006), that qualitative research process inevitably involves not just the participants’ but also the researchers’ ideas, thoughts and feelings, we believe that research coherency and rigour can be established through an investigator triangle (Hales, 2010). All themes have been checked and agreed upon by all researchers, and later by all participants.

#### Familiarisation

Interview data was read repeatedly in order to formulate initial interpretations, patterns, and themes (Bird, 2005; Braun & Clarke, 2006; Braun & Clarke, 2012).

#### Generating initial codes

Coding is conducted in order to begin the systematic data analysis, allowing to provide a label for different data features (Braun & Clarke, 2012). The coding process in this study was ‘theory-driven’ (Braun & Clarke, 2006), with the following research questions in mind:

The 35 codes from the interview dataset are presented as follows: (1) Positive emotions when finding out about having triplets, (2) Last chance to have children, (3) Confidence about childbirth, (4) Concern and worry from other people, (5) Judgment about having more than one baby, (6) Financial and domestic worries, (7) Feeling ‘full of baby’, (8) Mother’s feelings of boredom and restriction, (9) Receiving family support, (10) Feeling guilty about other children, (11) Having a big family as a joyous experience, (12) Positive mindset, (13) Support from nurses, (14) Being happy about healthy babies, (15) Accepting help, (16) Not allowing bad statistics impact your experiences, (17) Triplet personality differences, (18) Concern when finding out about having triplets, (19) Concern about mother’s health, (20) Encouraging triplets to be individual, (21) Positive experience of growing up with many siblings, (22) Support from family and friends, (23) Being treated differently by others due to having triplets, (24) Feeling scared by doctors about having triplets, (25) Lack of psychological support for postpartum depression, (26) Recognising each other’s strengths as a couple, (27) Feelings of inadequacy as a father, (28) Poor communication from medical staff, (29) Feeling that something is abnormal, (30) Closeness between triplets, (31) Feeling like a ‘human incubator’, (32) Providing a holding environment, (33) Seeing parenting as a relationship rather than a job, (34) Using Internet and Facebook to find other triplet parents, (35) Feeling special as parents of triplets.

#### Searching for themes

Using Braun and Clarke’s (2006) mind map process, each code was attached to a theme-pile to categorise codes at a broader level of data analysis. The 35 codes were categorised into six themes: Theme 1: Negative experiences of raising triplets prenatally and postnatally; Theme 2: Positive experiences of raising triplets prenatally and postnatally; Theme 3: Social, psychological, and material support; Theme 4: Experiences and challenges specific to mothers of triplets; Theme 5: Experiences and challenges specific to fathers of triplets; Theme 6: Advice for future parents of triplets. No codes were identified as an outlier.

#### Reviewing themes

Once the distinct themes were established, a second stage review process was conducted to check whether the themes relate to the entire data set (Braun & Clarke, 2012). Codes were re-read alongside the collated extracts (available in ‘Results’ below) to further refine the differences between the six themes (Braun & Clarke, 2006). The set themes were checked in accordance with the following criteria: they capture the most important elements of the data and they are relevant to the outlined research questions (see Table 2) (Braun & Clarke, 2012). The data were organised in the following manner: negative experiences (physical, social, psychological, material) that are specific to raising triplets prenatally and postnatally (corresponding to R1, Table 2); positive experiences (physical, social, psychological, material) that are specific to raising triplets prenatally and postnatally (corresponding to R3, Table 2); support networks that were identified as important and/or beneficial in parenting triplets (corresponding to R2, Table 2); experiences that are specific to mothers and fathers, which allowed us to draw significant variations between these two parent roles (corresponding to R4, Table 2); and finally, factors that could be beneficial for future parents of triplets (corresponding to R5, Table 1).

**Table 2:** Research questions

R1	What are the unique challenges of raising triplets prenatally and postnatally?
R2	What were useful in overcoming the parental challenges?
R3	What are the positive experiences of raising triplets?
R4	What are some of the differences between mothers’ and fathers’ experiences of parenting triplets?
R5	What advice would be useful for future parents of triplets?

#### Defining and naming themes

Collated data extracts were refined to ensure that each theme is consistent with the accompanying narrative (Braun & Clarke, 2012).

## Results

Our thematic analysis identified six themes: Negative and positive experiences of raising triplets prenatally and postnatally (Themes 1 and 2), useful support in raising triplets (Theme 3), experiences and challenges specific to mothers and fathers (Themes 4 and 5), and advice for future parents of triplets (Theme 6).

The data sets from Theme 1: Negative experiences of raising triplets prenatally and postnatally, revealed parents’ struggles in adjusting social, psychological, and material aspects of their lives to accommodate the triplets’ needs. Unsurprisingly, these adjustments are experienced as intense and demanding in multiple births, often requiring drastic changes in parents’ careers as well as domestic and social lives. In contrast, Theme 2: Positive experiences of raising triplets prenatally and postnatally, demonstrated parents’ happy moments, both prior and after birth. Many positive expressions and experiences relate to the idea that triplets are a special and rare phenomenon, which is interesting for many people outside of the family circle. In Theme 3: Social, psychological, and material support that was useful in parenting triplets, parents revealed the different support networks that were useful both prior and after the birth of triplets. Theme 4: Experiences and challenges specific to mothers of triplets, and Theme 5: Experiences and challenges specific to fathers of triplets, draw differences between fathers’ and mothers’ experiences of parenting triplets. Finally, Theme 6: Advice to future parents of triplets conveys some of useful tips on how to overcome the challenges of parenting triplets prenatally and postnatally, such as allowing themselves to accept support.

### Theme 1: Negative Experiences of Raising Triplets Prenatally and Postnatally

All study participants revealed that they had immediate financial and domestic concerns once they learned that they are having triplets.

*Participant 2:* First emotion was [that of] a concern because it’s an unusual situation. Economically, this means it’s another three children. [It adds to] financial and medical concerns.

*Participant 6:* We came out in car, broke into tears. The news was great, being pregnant is wonderful, but being pregnant with triplets was… massive. … How are we going to afford everything, cope financially, [and] not give up jobs and still care for children?

*Participant 7:* I was so shocked. … I felt emotionally overwhelmed, a bit like, “oh my god, I just can’t believe it”. Day after that, I panicked, and I was afraid for a long time, thinking if [the triplets] were going to be okay. […] I wouldn’t have money to support them, [and] I had those fears all the time.

Many of these concerns were coupled with health worries relating to both mothers and triplets. Several participants noted that the initial communication from doctors and other medical staff was unclear and made the parents feel as if something was wrong or that there was an abnormality with the pregnancy.

*Participant 3:* At the 8^th^ week, doctor told us that we are having twins, but I felt that is manageable. At the 12^th^ week, they said we are having triplets. That was shocking. As a working nurse myself, I knew a lot of statistics associated with risks [of having triplets]. I was happy with being pregnant, but anxiety started to grow, worried about babies’ and my health. Worried about whether they will be born healthily.

*Participant 5:* My husband and I went to the hospital and got a scan. They asked if I have taken fertility drugs, [and I said] no, of course not. Radiographer then told us that she sees three bodies. I said: “Pardon?” My first thought was abnormality. Three babies? She got somebody else to check. I was upset, I wasn’t prepared, and we were both worried. What about complications?

*Participant 6:* As young parents, we were worried. The radiographer brought a colleague in and said we should get a senior radiographer. They were talking to each other but saying nothing to us. Then, the senior radiographer said, “oh my god, we can see three heads”. At that time, we thought we have one baby with three heads. They haven’t explained it well at all. For scared people who are not familiar with the [medical] system, it was poor communication. It set up negative feelings that there is a problem or something that needs to be sorted out.

For a family with another sibling, care for the mental health of that child is another important issue.

*Participant 4*: All of our [his and his wife’s] attention had to go to the triplets, so our daughter felt our love for her was stolen. It seems to me that she has become more frequently angry, and sometimes she behaves like a baby. We weren’t, probably still aren’t, certain how to approach her. We have just been trying to have a one-on-one time with her.

Finally, many participants noted the physical exhaustion and difficulties in managing time after the birth of the triplets.

*Participant 2:* [There] is a huge change in your life after birth. The ability to go to a park for a walk is now [replaced] with feeding and wrapping the babies and getting them into car. […] All out of sudden, there are other things to think about. I couldn’t just flip myself and say, “I will be there at 8 o’clock in London.” I may not get enough sleep, so getting a 6:30am train was not even an option.

*Participant 8:* I was at Uni, working full-time. I remember this excruciating feeling of “why can’t they just have a day off?” […] Babies get up at 4am, and “it’s your turn, my turn” kind of argument [with my wife]. Saturday morning, I want to be slow, but they are up, and there is no slow time.

Financial concerns, health worries, physical exhaustion and limited time were noted as primary negative experiences by the participants.

### Theme 2: Positive Experiences of Raising Triplets Prenatally and Postnatally

One participant was particularly positive and confident about giving birth to triplets. Differently from most participants, the dataset revealed that one couple in particular struggled to have children and had to undergo fertility treatment with their firstborn. This has made the mother feel more confident and positive despite negative statistics associated with multiple births:

*Participant 1:* It was my last chance to have children. My husband was worried; we had to undergo fertility treatment with our [firstborn], and I didn’t want to get the treatment again. I wanted to have four children, and I had a massive feeling that it will be alright. We were astounded when we heard the news. Immediately, the [medical staff] told the scary stats. They pushed, but I took the risk.

Two participants referred to the importance of having (and being part of) a big family as a positive factor in parenting triplets.

*Participant 1:* We created a big family, and that’s why our mindset was positive. […] Having them was busy but it was also so joyous. They were healthy, and nurses felt privileged to be there, [witnessing] three healthy babies. It was a novelty for them, it brought so much happiness.

*Participant 2:* I had a positive experience being raised with four siblings. […] The shift toward becoming family-focused was natural. […] We’ve been lucky with all the kids. They have an effect on everyone, everyone loves them. You really think, “wow, this is what it means to have a family”.

Lastly, several participants noted the special bond between triplets, which has been likened to witnessing a unique phenomenon both by parents and strangers outside of the family circle.

*Participant 6:* The triplets were communicating and laughing between themselves. I remember those mornings, taking a picture of them, it was amazing. We were listening to them and bringing them to bed, and they would all go to bed at the same time. Some real wonderful experience in the early days. They learn the concept of sharing very early on, and do things together.

*Participant 8:* We became famous. When we left [our town], the security and people were smiling, crowding around us. […] People were asking questions even if they don’t know you. […] [Life] with triplets becomes funny. When triplets used to crawl, I would lie in the living room, and they’d crawl all over me, in circle, same direction. It was magical and fun.

Participants reported a sense of a big family, and strong bonds among themselves as positive experiences.

### Theme 3: Social, Psychological, and Material Support

All participants noted the importance of having support from the medical staff and hospital, both prenatally and postnatally. This includes not just support for mothers’ and triplets’ physical health and wellbeing but also psychological support.

*Participant 5:* My care was amazing. Medical staff were very careful, sensitive, and nice. I had lots of scans, and I developed a great relationship with the consultants. There was a team for each baby. […] I was in a special care unit, which also had [cared] for postnatal depression. It could be because of the separation factor [since] the triplets were in an incubator. It was a strange emotional [experience].

*Participant 7:* My girls were born in private hospital. I felt relaxed, they were well looked after, had a room just for them. There were three incubators, I could sit and be with them. [I was not] able to hold them yet because they were too small.

Two other factors that played an important role in overcoming parenting-related challenges were financial support and help from family and friends.

*Participant 6:* Never had any negative reactions. Everyone was lovely: family, friends, neighbours. They ensured we had everything we needed. Just knocked on the door, and they were lovely. Very few people gave guidance. […] Our circle of friends grew. We didn’t lose anybody. All friends, same age, with babies, we got to know people who are also multiple birth parents.

*Participant 8:* At the most basic, what helps is having a supportive partner. I’m sure it’s the other way around too. Also having [family] support, that’s huge help. We received financial help from my [partner’s] family. The triplets got beautiful clothes and were well dressed. […] With financial support, we were able to go out as a family with our babies.

Some participants highlighted the value of external support by referring to an experience where they did not have such support.

*Participant 4*: We were kind of hoping that our parents will come to help us. My mother in-law came to help us for a few days, but she didn’t agree with how we take care of triplets, so had an argument with my wife, and left. We were left alone to take care of triplets and a four-year-old. As we had hope up, it was physically and mentally challenging to adjust to the reality that we are alone.

*Participant 8:* You don’t get things for free, but my hope is that people will realise that going from one to three children is something rare and expensive, and everything comes in three at the same time. [I remember] when we flew back from London, we needed help getting off the plane, but no one helped for 45 minutes. Eventually, someone helped us from the airline. On the other hand, when we flew on Christmas, [the staff] took care of all babies, for ten minutes they walked around with the [staff], while we got breakfast.

Participants reported that more support is needed for parents of triplets, and the felt value of such support was immense to them.

### Theme 4: Experiences and Challenges Specific to Mothers

Interviews with mothers included many expressions and thoughts that pertained to experiences of pregnancy (i.e., of *being* and *feeling* pregnant) with triplets. In comparison to interviews with fathers, mothers frequently discussed their prenatal and postnatal experiences as internal rather than external, without necessarily clearly differentiating between themselves and the babies (i.e., referring to a mother-baby unit).

*Participant 7:* I was a human incubator for babies. I was there for them. I struggled with my hormones a lot, I felt nervous. If someone was annoying me, I would rush out easily. I felt I had to deal with my emotions, to be calm and provide for the babies […] because I knew it would have an impact on them.

*Participant 1:* My mom was worried, my sister was confident just like me. Most people said, “oh my god” or “as long as you have one baby”. But I was happy. […] I felt full of baby.

### Theme 5: Experiences and Challenges Specific to Fathers

The dataset from interviews with fathers of triplets revealed feelings of inadequacy and discomfort when witnessing mothers’ physical and psychological pain associated with pregnancy.

*Participant 6:* For me as a male, trying to make sure [the mother] is as comfortable as possible was crucial. I felt completely impotent; as a man, I can’t carry children, and it was tough for the mother, she was in lots of pain.

*Participant 2:* There were a couple of instances where [the mother] was not particularly well. Five months in, she had incredible headaches. Migraine type. From my point of view, I was always concerned about her.

In comparison to interviews with mothers, fathers more frequently stressed financial and domestic concerns, and work pressures.

*Participant 2:* It was very difficult pressure workwise. I am an engineer, and for the past 25 years, I worked in sales […] in a competitive US company. There [was] additional pressure to carry on being successful at work with triplets at home.

*Participant 8:* The triplets went to a [private school], we had to negotiate to pay two and a half fees. In another country where they went to school (father in-law paid), we had to strike a deal with them again, and pay two and a half fees.

*Participant 4*: When we found out we are having triplets, I thought I should quit my job to help my wife. But then we won’t be able to survive financially. … [After triplets were born] I worked really hard, but on reflection I sometimes feel regretful that I could have taken more days-off back then to support my wife.

### Theme 6: Advice for Future Parents

Interestingly, the main advice from all participants was to learn how to accept different forms (e.g., financial, social, physical) and sources (e.g., from family members, public services, hospitals) of help. This implies that there may be an initial psychological difficulty or anxiety felt by new parents of triplets in being supported by friends or family, as well as discomfort in receiving support.

*Participant 1:* Stay calm, don’t look too far into the future. Try to enjoy. Accept as much help as possible.

*Participant 5:* Try not to be too anxious. People offer help, just accept help. Look out for the community and friendships, not just for yourselves as parents but also for children. See this as a social experience.

*Participant 3*: I have become less ashamed to express my emotional distress to my friends, “I am stressed”, “I’m depressed” and so on. Then they suggest we go to the park or shopping together. This was helpful. I found that not being alone, being with someone very helpful.

Other advice included finding other people with multiple birth experiences, recognising each other’s strengths as partners and parents, being part of a supportive community, and meditation.

*Participant 2:* Try to find people who had similar experience or are interested in triplets. When [my partner] told me about this study, she was excited. First thing I said was that I wish we had known someone [like us] at the time. As much as your immediate family supports you, someone who has gone through can give you better advice. So, first bit is to find someone with same experience, whether virtually, on social media, etc.

*Participant 5:* Recognise each other’s strengths. It’s easy to blame partners. […] Try not to be resentful, communicate; it’s okay to be angry, but you need to talk about it. We could have easily divorced but we went through this because we were on it together.

*Participant 5: Y*ou have to be in a community […] instead of closing off others from yourself. Respecting other people, but also [inviting] them to your [space].

One participant reported a specific intervention to address stress.

*Participant 7:* Meditation. People struggle all the time. I wanted to have a profession, to bring money in… But you don’t have time when raising children, you are so busy with three babies, then it all starts to hit you. […] Meditation really helped me. I felt serene. I am now a Buddhist myself, […] I focus on meditation, breathing, and self–monitoring.

Acceptance of support and stress reduction exercises such as meditation were noted as advice for future triplet parents.

## Discussion

This study qualitatively analysed semi-structured interviews with eight parents of triplets (four male-female couples), reflecting on their pre- and post-natal experiences. Six themes were identified: Negative and positive experiences of raising triplets prenatally and postnatally (Themes 1 and 2), useful support in raising triplets (Theme 3), experiences and challenges specific to mothers and fathers (Themes 4 and 5), and advice for future parents of triplets (Theme 6).

One original contribution of this study was an appraisal of the first-hand experiences in parents of triplets to address the lack of mental health care for parents of triplets (Bricker, 2014). Consistent with previous triplet studies (Baor & Blickstein, 2005; Mhatre & Craigo, 2020), our participants reported experiencing mental distress associated with psychological and financial impact of having triplets, and health concerns of arriving babies and the mother. Emotional words such as ‘anxious’, ‘worried’, ‘stressed’, ‘panicked’, and ‘excruciating’ were often used to describe the psychological status during the prenatal and postnatal phases. These pieces of data can further illustrate the emotional life of parents, elucidating the previously identified risk factors in parents of triplets, such as reduced quality of life (Roca-de-Bes et al., 2011) and emotional exhaustion (Mhatre & Craigo, 2020). As recommended by Roca-de Bes et al. (2011), if psychotherapy is used for parents of triplets, intervention targeting these emotions may be particularly effective. For example, self-compassion interventions have been reported effective addressing stress, anxiety and emotional exhaustion in parents (Mitchell et al., 2018; Robinson et al., 2018; Sirois et al., 2019). Considering their limited time, online intervention may be more feasible (e.g., Michell et al., 2018). Future research needs to evaluate the effect of this type of intervention on parents’ mental health.

Our results also revealed that, after the birth, parents had difficulties forming a firm bond with each child, as reported previously (Akerman, 1999; Albrecht & Tomich, 1996; Leonard, 1998). Additionally, our analysis identified that in a family where there is another sibling, maintaining a strong relationship with the child can be also difficult, because (i) all the parental attention is focused on newly born triplets, and (ii) the older child experiences significant reduction of received attention after the birth. Care is needed for these family members too, suggesting the importance of inter-sector care for families of triples, involving hospital, school, and communities.

Another unique contribution is that this study compared mothers’ and fathers’ parenting experiences (Themes 4 and 5). Mothers’ challenges were generally described as *internal*, concerning their and triplets’ physical and psychological health and wellbeing, which aligns with previous research (Bricker, 2014). This finding also corresponds with theory and research conducted earlier by paediatrician and psychoanalyst Donald Winnicott, who famously observed that the ‘mother-baby unit’ is inseparable in the first years of infant’s life, during which maternal care (comprising physical and psychological needs) enables the infant to develop despite their lack of control over external environment (Winnicott, 1960). For Winnicott, the holding environment created by the ‘mother-baby’ unit, facilitates a form of self-soothing and fosters a sense of increasing interdependence in children. The responses in our study suggest that the larger interdependent unit with three babies may also foster a unique sense of *sharedness, separateness* and *holding* between the triplets and their mothers. Further research is needed in the fields of infant development, which have historically focused on single child development and sibling conflict.

Contrarily, fathers’ experiences were more related to *external* factors such as their job and finances. Indeed, the health risks are high and thus are often the main concern in triplet pregnancy (Blumenfeld et al., 2008; Bricker, 2014); however, fathers’ role in this domain is limited. The limited purview of the internal domain may force fathers to focus on the external domain including jobs, income and other economic provisions needed to take care of a large family. Though our findings are difficult to be generalised, these differences may be helpful when healthcare workers engage with mental health difficulties in parents of triplets. For mothers, attachment-based approaches may be more appropriate, centred around the connections between them. On the other hand, more solution-focused approaches (Kotera, 2018) may be more effective for fathers, considering a variety of life domains to identify actions. As seen in other contexts (e.g., Tadic et al., 2019), gender-specific approach may be helpful when caring for parents of triplets.

Lastly, helpful recourses and advice for future parents of triplets (Themes 3 and 6) can offer practical and helpful information, with self-compassion as one approach to protect their mental health. While triplet research so far has focused on risks and challenges associated with delivering and raising triplets, our analysis identified what is helpful for parents of triplets, which can help develop psychological resources such as resilience and self-compassion (Kotera et al., 2020). Indeed, one participant received preventative treatment for depression, which was found helpful. To counter mental health difficulties, social support from family members and friends was noted as highly helpful. Social support is associated with a high level of resilience, which is closely related to self-compassion (Kotera et al., 2021), helping individuals to overcome challenges (Ozbay et al., 2007; Southwick et al., 2016). Further, our participants reported that a sense of reassurance was important to cope with stress and anxiety prenatally and postnatally. This is in line with mental health studies that self-compassion, a similar construct to self-reassurance, was identified as strong predictor of better mental health (Kotera et al., 2019, 2021). Self-compassion and self-reassurance can activate our soothing system, as opposed to the threat or drive systems in our mind, leading to better mental health (Gilbert, 2010). Meditation, an intervention often used to cultivate self-compassion (Kotera, 2021), was reported helpful. Lastly, participants reported that accepting support from others is advisable for future parents of triplets. Indeed, individuals with self-compassion are more likely to be able to accept support from others (Gilbert et al., 2011, Kotera et al., 2021). Taken together, as suggested above, targeting self-compassion may be an effective means to support the mental wellbeing of parents of triplets. Practice and research need to be implemented.

### Limitations

While this study offers helpful findings, several limitations need to be noted. First, the findings are identified from the current small sample of parents of triplets, consisting only of intact families (e.g., non-divorced parents). As such, these findings may not apply to the general population of parents of triplets. Though recruiting parents of triplets is difficult due to the limited number of samples and limited time they can devote to research, more diverse samples should be recruited to develop more generalisable findings. Relatedly, one couple in our study was from Japan, while the remaining three were from the UK. Although we did not find any significant difference in data, considering the cultural differences in parenting (Bornstein, 2015), more data may enable cross-cultural comparisons. Additionally, this study did not include parents of triplets who encountered serious health problems; all triplets have been developing healthily and/or typically in this study. Given the high rates of complications, parents of triplets who underwent serious health problems or disabilities/deviations should be included in future research. Lastly, we did not consider the socioeconomic background or larger family structure of the participants. As these were indicated to be relevant in our analysis, future studies should include these aspects.

## Conclusion

The number of parents of triplets and the demand for resources to support them are on the rise, however, targeted support for parents of triplets remains to be evaluated. Although our study features a small sample, we were able to identify first-hand experiences of parents of triplets, and possible interventions to support their wellbeing, which correspond to unique idiosyncratic experiences revealed through semi-structured interviews. Our findings will help parents of triplets, healthcare workers and communities to develop effective means to reduce the mental health difficulties that this under-researched population faces.

## Supplementary information

Appendix 1 Triplet Study

## Data Availability

The datasets generated during and/or analysed during the current study are available from the corresponding author on reasonable request
