# Supplementary material for: Qualitative Investigation into Pre- and Post-Natal Experience of Parents of Triplets
Source: J Child Fam Stud. 2021 Dec 18;31(7):1785–97. doi: 10.1007/s10826-021-02200-1 (PMC8683308; doi:10.1007/s10826-021-02200-1)
Supplement: Supplementary file 1 — Appendix 1 Triplet Study [file 10826_2021_2200_MOESM1_ESM.docx]

Appendix 1

**Interview Schedule**

The aim of this interview schedule is to explore your pre- and post-natal experience of triplets as a parent. Below are guide questions to stimulate this explorative discussion.

*What did you feel when you first found out that you were having triplets?*

*Any thoughts on people’s reactions when you told them that you were having triplets?*

*What was most challenging pre-natally and post-natally?*

*What did you find useful to overcome those challenges?*

*What were the positive impacts of having triplets pre-natally on other areas of your life?*

*What have been the positive impacts of raising triplets post-natally on other areas of your life?*

*Any advice for future parents of triplets?*

*Is there anything else that you feel I should have asked, or that you would like to add?*

[Researcher’s contact information]
